# Apgar Score Is Related to Development of Atopic Dermatitis: Cotwin Control Study

**DOI:** 10.1155/2013/712090

**Published:** 2013-10-09

**Authors:** Vibeke Naeser, Niklas Kahr, Lone Graff Stensballe, Kirsten Ohm Kyvik, Axel Skytthe, Vibeke Backer, Charlotte Giwercman Carson, Simon Francis Thomsen

**Affiliations:** ^1^Department of Respiratory Medicine, Bispebjerg Hospital, 2400 Copenhagen, Denmark; ^2^Danish Epidemiology Science Centre, Statens Serum Institut, 2300 Copenhagen, Denmark; ^3^Institute of Regional Health Services Research & Odense Patient Data Explorative Network, University of Southern Denmark, 5000 Odense, Denmark; ^4^The Danish Twin Registry, University of Southern Denmark, 5000 Odense, Denmark; ^5^Danish Pediatric Asthma Center, Gentofte Hospital, 2900 Hellerup, Denmark; ^6^Department of Dermato-Allergology, Gentofte Hospital, 2900 Hellerup, Denmark

## Abstract

*Aim*. To study the impact of birth characteristics on the risk of atopic dermatitis in a twin population. *Methods*. In a population-based questionnaire study of 10,809 twins, 3–9 years of age, from the Danish Twin Registry, we identified 907 twin pairs discordant for parent-reported atopic dermatitis. We cross-linked with data from the Danish National Birth Registry and performed cotwin control analysis in order to test the impact of birth characteristics on the risk of atopic dermatitis. *Results*. Apgar score, OR (per unit) = 1.23 (1.06–1.44), *P* = 0.008, and female sex, OR = 1.31 (1.06–1.61), *P* = 0.012, were risk factors for atopic dermatitis in cotwin control analysis, whereas birth anthropometric factors were not significantly related to disease development. Risk estimates in monozygotic and dizygotic twins were not significantly different for the identified risk factors. *Conclusions*. In this population-based cotwin control study, high Apgar score was a risk factor for atopic dermatitis. This novel finding must be confirmed in subsequent studies.

## 1. Introduction

Atopic dermatitis is a chronic relapsing skin disease with a lifetime prevalence of around 20%. The disease has an early onset with 60% being affected during the first year of life and 85% before the age of five [1]. Atopic dermatitis is characterized by recurrent episodes of itching and eczema occurring at typical sites such as the face and extensor aspects of the arms and legs in early childhood, flexures in later childhood, and head and neck as well as the hands in adulthood. Atopic dermatitis is highly heritable and individual susceptibility to the disease is attributable particularly to mutations in the filaggrin gene [2], although other genetic variants [3] as well as environmental factors have been implicated. Previous studies have shown that the prevalence of atopic dermatitis has increased both in developed [4, 5] and developing countries [6] in recent years, but the causes for this increase are still poorly understood.

The onset of atopic dermatitis early in life implies that risk factors for the disease exert their effect already in utero or in very early childhood. However, previous studies of the association between perinatal factors and the risk of atopic dermatitis are conflicting. With regard to alcohol intake during pregnancy, results indicate an increased risk for atopic dermatitis in children of mothers who consume alcohol during pregnancy [7, 8]. Contrary to this, smoking during pregnancy has been inversely related to development of atopic dermatitis in the offspring [9, 10]. The association between exclusive breastfeeding/increased duration of breastfeeding and atopic dermatitis varies; while some studies show a positive association [11] others show a negative [12] or no [13] association. Some studies [9, 14] but not all [15] have found that high birth weight increases the risk of atopic dermatitis, whereas other studies have found that a higher gestational age increases the risk of atopic dermatitis [16]. Mallen et al. investigated the association between six birth-related exposures (birth weight, mode of delivery, artificial commencement of labor, prematurity, neonatal intensive care unit admission, and fetal distress) and asthma, allergic rhinitis, eczema, and hay fever in adulthood [17]. No statistically significant association was demonstrated between any of the birth-related exposures and the four allergic conditions studied, although some nonsignificant trends were noted for those born by Caesarean section and those exposed to fetal distress during labor.

The cotwin control study design is a matched case-control study that utilizes twin pairs discordant for an exposure or an outcome. Particularly, because twin pairs are inherently matched on several confounding factors relating to early life and upbringing, this type of study design is ideal to tease out causative relationships in complex diseases. In the present study we use the cotwin control design to identify birth-associated risk factors for atopic dermatitis in twin pairs discordant for the disease.

## 2. Methods

The study population comprised all live-born twins in Denmark between 1994 and 2000 included in the Danish Twin Registry. In 2003 when the twins were 3–9 years of age the twins' parents responded to a two-page questionnaire concerning atopic diseases, diabetes, febrile and epileptic seizures, and mental health attributes. Atopic dermatitis was diagnosed by the question “has your child ever had eczema in the folds of the elbows or knees?” Zygosity was determined by information regarding the twins' similarity to family, friends, teachers, and relations. This method determines zygosity correctly in more than 95% of the cases compared with genetic marker information [18]. We cross-linked the information from the questionnaire with data from the Danish National Birth Registry where data on birth weight, birth length, maternal smoking during pregnancy, gestational age, mode of delivery (Caesarean section versus vaginal delivery), and Apgar score were obtained. 

A total of 10,809 twin subjects participated in the questionnaire study (participation rate 68%). Of these, complete information on atopic dermatitis was available for 5399 intact twin pairs: 850 monozygotic (MZ) twin pairs, 2,279 dizygotic (DZ) twin pairs of the same sex, 1,999 DZ twin pairs of opposite sex, and 271 twin pairs of unknown zygosity. There were 907 twin pairs discordant for atopic dermatitis, and of these 58 and 827 were monozygotic (MZ) and dizygotic (DZ) pairs, respectively. 

### 2.1. Statistical Analysis

**Figure 1:**
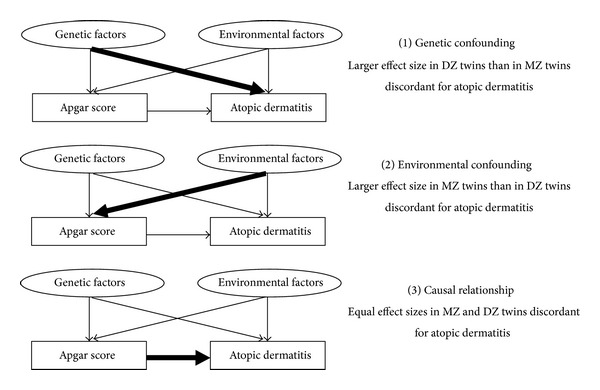
Possible association between Apgar score and atopic dermatitis predicted by the cotwin control method.

First, the relationship between birth-associated risk factors and atopic dermatitis was examined in the whole population using unpaired *t*-tests and chi-square tests, ignoring the familial relationship between the twins. Next, the risk factors that were unshared between the twins within the pair were examined in a 1 : 1 matched conditional logistic regression analysis (cotwin control analysis) in order to determine the effect of these risk factors on atopic dermatitis. The factors entered into this analysis were birth weight, birth length, Apgar score, and sex. The matching was done with the affected (atopic dermatitis) twin in each pair being the case and the unaffected twin being the control. DZ same-sex and DZ opposite-sex twin pairs were considered together in the analysis. The matching of the twins in the cotwin control design allows an inherent adjustment for other factors that would otherwise confound the association between birth factors and atopic dermatitis such as genetic factors, atopic predisposition, maternal smoking during pregnancy, gestational age, mode of delivery, and current age, as well as effects of the rearing environment. Therefore the estimated risk of atopic dermatitis can be assumed to reflect more precisely the influence of birth factors, for example, Apgar score. The following predictions can be made for the cotwin control analysis (Figure 1): if the association between birth factors and atopic dermatitis is mediated via genetics, then we do not expect to find any significant risk of atopic dermatitis among discordant MZ twin pairs, because MZ twins are concordant for early rearing environmental and genetic factors. Alternatively, an increased risk of atopic dermatitis in discordant DZ twin pairs would suggest that genetic factors underlie the association, because DZ twins share the same rearing environment, but only half their genetic variants. Finally, a direct (causal) effect of birth factors, for example, Apgar score, on atopic dermatitis would be suggested by the finding that both discordant MZ and DZ twin pairs are also discordant on, for example, Apgar score, as the association can neither be reflected by environmental nor genetic factors [19]. Data were analyzed with the statistical package SPSS 18.0 (SPSS Inc., Chicago, IL).

## 3. Results

The proportions of girls and boys in the population were 48.9% and 51.1%, respectively. The mean age was 5.9 years with no difference between girls and boys. The overall prevalence of atopic dermatitis was 14.9%, 14.0% in boys and 16.0% in girls, *P* = 0.003. Increasing birth length (*P* = 0.038), nonsmoking of the mother during pregnancy (*P* = 0.002), and monozygosity (0.012) were also significant predictors for atopic dermatitis in the whole sample when ignoring the familial relationship between the twins (Table 1).

 Within twin pairs discordant for atopic dermatitis, the affected twin had, on average, a higher Apgar score and was smaller at birth compared with the unaffected twin (Table 2). In multiple conditional logistic regression comparing the affected with the unaffected twin, Apgar score, OR (per unit) = 1.23 (1.06–1.44), *P* = 0.008, and female sex, OR = 1.31 (1.06–1.61), *P* = 0.012, were significantly predisposing factors for atopic dermatitis, whereas birth anthropometric factors were not significantly related to development of atopic dermatitis. Risk estimates in discordant MZ and DZ twins were not significantly different (Table 3).

## 4. Discussion

In this nationwide cohort of Danish twin pairs discordant for atopic dermatitis, a high Apgar score and female sex were significant determinants of atopic dermatitis, whereas birth weight and birth length were not significantly related to disease development.

Apgar score is a clinical test performed on a newborn one and five minutes after birth. It is a composite measure of breathing effort, heart rate, muscle tone, reflexes, and skin colour and is an indicator of the newborn's need for medical attention shortly after birth. A detrimental effect of high Apgar score on the risk of atopic dermatitis is, to our knowledge, a novel finding that needs to be confirmed in subsequent studies. Among Finnish children low Apgar score increased the risk of asthma [20, 21]. Contrary to this, Apgar score was not related to asthma [22] or hay fever [23] in Finnish twin children and did not interact with in vitro fertilization on the risk of asthma in children born after assisted reproduction [24]. Moreover, Gilman et al. found no significant association between Apgar score and maternal smoking [25]. It is somewhat counterintuitive that it is the twin with the highest Apgar score that has the highest risk of later development of atopic dermatitis. However, previous studies have found a harmful effect on atopic dermatitis of other birth-related factors linked to high Apgar score such as increased fetal growth [9], high socioeconomic status (SES) [26, 27], and nonsmoking of the mother [9, 10]. Particularly, Lundholm et al. examined the association between fetal growth and atopic diseases and found that the rate of atopic dermatitis increased with higher levels of the mother's education and furthermore that children of mothers who did not smoke during pregnancy also had slightly higher rates of atopic dermatitis compared with children of mothers who smoked during pregnancy [9]. Furthermore, a Dutch study showed that smoking during pregnancy resulted in decreased birth weight and that women of low SES give birth to lighter babies [28]. These findings are inline with the assumption that atopic dermatitis is a disease affecting apparently robust children from high SES families. Because the affected (atopic) and the unaffected twin were matched on exposure status in our paired analysis, the observed positive association between atopic dermatitis and Apgar score is adjusted for confounding due to SES, parental smoking, and gestational age. Taken together, multiple factors are involved in the development of atopic dermatitis, particularly, genetics, fetal programming, and environmental effects, which makes it difficult to deduce which risk factors represent direct effects and which represent mediatory effects. The equal effect sizes in MZ and DZ twins for Apgar score in cotwin control analysis suggest a direct effect of one or more of the factors attributable to the Apgar score in relation to atopic dermatitis.

The prevalence of atopic dermatitis was higher in girls than in boys in the present study. Other studies contradict this, concluding that boys have a higher prevalence of atopic dermatitis and yet other studies find no significant difference between the prevalence of atopic dermatitis in boys and girls. Notably, a study from Taiwan found that the prevalence of atopic dermatitis in females is lower than in males before the age of 8 years but subsequently becomes higher [29], whereas studies from Japan and Italy show no significant difference in the prevalence of atopic dermatitis in boys and girls [30, 31]. A recent study of another (comparable) Scandinavian population from Sweden found a female preponderance of atopic dermatitis (14.5% in girls versus 9.4% in boys) [32].

We found an overall higher prevalence of atopic dermatitis among the entire group of MZ twins than among the group of DZ twins. This finding could be due to confounding from factors in the MZ group not present in the DZ group, for example, a larger susceptibility gene pool among MZ twins or a more unfavorable intrauterine environment in MZ than in DZ twins. Also, these could be factors such as differences in the MZ and DZ families in terms of smoking and SES, but evidence in support of this theory is lacking. 

The large population size with identification of 907 twin pairs discordant for atopic dermatitis and the high response rate are strengths of this study, although the number of discordant MZ pairs was quite small (58 in total). We had complete ascertainment of all live-born twins in Denmark between 1993 and 2000 and cross-linked these data to data from the Danish Birth Registry combining information from two independent sources. Data on perinatal risk factors were obtained by professional midwifes' records, which increased the validity of the data. Contrary to this, the diagnosis of atopic dermatitis was self-reported by the parents of the twins, which could have induced recall and reporting bias. Moreover, the question used to diagnose atopic dermatitis has not been systematically validated. Another limitation alludes to the differing age at ascertainment of the eczema diagnosis, which may have influenced the results. However, age was automatically eliminated as possible confounder in the cotwin control analysis model since it was the same for the two twins within the pair. 

The fact that we studied twins may have had implications for the generalisability of the results to the population as a whole. Particularly, twins differ from singletons in a number of ways related to prenatal and early life that potentially could affect the risk of atopic dermatitis differentially in twins. These factors, which may be related to gestation, intrauterine environment, and mode of delivery, could also be expected to influence the variation in Apgar score. However, in a matched case-control study of 112 infant twins and singletons from the United States 5-minute Apgar score was not different in twins compared with singletons when matched for gestational age and mode of delivery [33], suggesting that results in relation to Apgar score can be extrapolated to the general singleton population. Still, we acknowledge that there may be unmeasured factors in our sample that could have interfered with our conclusions. 

We conclude that a high Apgar score may be linked to development of atopic dermatitis. Although this finding agrees with the theory that robust babies of high SES are at increased risk of atopic dermatitis, this has not been previously documented and awaits support from future studies.

## Figures and Tables

**Table 1:** Characteristics of subjects with and without atopic dermatitis in a sample of Danish twin pairs, 3–9 years of age.

	Atopic dermatitis		OR (95% CI)	*P* value
	Yes *N* = 1614	No *N* = 9195		
Birth weight, g	2593	2571	22 (−9–52)	0.167
Birth length, cm	48.24	48.05	0.19 (0.01–0.38)	0.038
Apgar score	9.76	9.73	0.03 (−0.02–0.09)	0.172
Gestational age, days	254.9	254.6	0.32 (−0.61–1.26)	0.496
Caesarean section	14.4	15.3	0.93 (0.84–1.04)	0.220
Maternal smoking	12.9	15.5	0.80 (0.70–0.92)	0.002
Sex				
Boys	14.0	86.0	1.00	
Girls	16.0	84.0	1.17 (1.05–1.30)	0.003
Age, years	5.92	5.86	0.06 (−0.05–0.17)	0.257
Zygosity				
DZ	14.4	85.6	1.00	
MZ	16.8	83.2	1.20 (1.04–1.38)	0.012

Means and proportions are calculated from available data for the individual variables.

OR is mean difference for birth weight, birth length, Apgar score, gestational age, and age.

**Table 2:** Distribution of perinatal risk factors for atopic dermatitis in 907 twin pairs, 3–9 years of age, discordant for atopic dermatitis.

	All twin pairs (*n* = 907)		MZ twin pairs (*n* = 58)		DZ twin pairs (*n* = 827)	
	Affected twins	Unaffected twins	Affected twins	Unaffected twins	Affected twins	Unaffected twins
Birth weight, g	2627	2649	2624	2625	2632	2650
Birth length, cm	48.37	48.41	48.60	48.17	48.40	48.44
Apgar score	9.81	9.68	9.80	9.75	9.80	9.67
Female sex	52.3	47.4	53.4	53.4	53.1	47.8

**Table 3:** Significant risk factors for atopic dermatitis in 907 twin pairs, 3–9 years of age, discordant for atopic dermatitis.

	All twin pairs		MZ twin pairs		DZ twin pairs		OR_MZ_ = OR_DZ_
	OR (95% CI)	*P* value	OR (95% CI)	*P* value	OR (95% CI)	*P* value	*P* value^1^
Birth weight	1.02 (0.81–1.28)	0.884	0.96 (0.12–7.86)	0.972	0.98 (0.77–1.24)	0.880	NS
Birth length	1.00 (0.08–13.03)	1.00	8.31 (N/A)	0.854	1.62 (0.11–22.01)	0.758	NS
Apgar score	1.23 (1.06–1.44)	0.008	1.19 (0.60–2.34)	0.615	1.21 (1.04–1.42)	0.015	NS
Female sex	1.31 (1.06–1.61)	0.012			1.31 (1.07–1.61)	0.011	NS

Only statistically significant predictors for atopic dermatitis are presented in the table (adjusted for birth weight, birth length).

^1^Test for heterogeneity between odds ratios for MZ and DZ twin pairs.

NS: nonsignificant.

## References

[1] Akdis CA, Akdis M, Bieber T (2006). Diagnosis and treatment of atopic dermatitis in children and adults: European Academy of Allergology and Clinical Immunology/American Academy of Allergy, Asthma and Immunology/PRACTALL consensus report. *Allergy*.

[2] van den Oord RAHM, Sheikh A (2009). Filaggrin gene defects and risk of developing allergic sensitisation and allergic disorders: systematic review and meta-analysis. *British Medical Journal*.

[3] Paternoster L, Standl M, Chen CM (2011). Meta-analysis of genome-wide association studies identifies three new risk loci for atopic dermatitis. *Nature Genetics*.

[4] Stensen L, Thomsen SF, Backer V (2008). Change in prevalence of atopic dermatitis between 1986 and 2001 among children. *Allergy and Asthma Proceedings*.

[5] Ninan TK, Russell G (1992). Respiratory symptoms and atopy in Aberdeen schoolchildren: evidence from two surveys 25 years apart. *British Medical Journal*.

[6] Asher MI, Montefort S, Björkstén B (2006). Worldwide time trends in the prevalence of symptoms of asthma, allergic rhinoconjunctivitis, and eczema in childhood: ISAAC Phases One and Three repeat multicountry cross-sectional surveys. *The Lancet*.

[7] Carson CG, Halkjaer LB, Jensen SM, Bisgaard H (2012). Alcohol intake in pregnancy increases the child's risk of atopic dermatitis. The COPSAC prospective birth cohort study of a high risk population. *PLoS ONE*.

[8] Linneberg A, Petersen J, Grønbæk M, Benn CS (2004). Alcohol during pregnancy and atopic dermatitis in the offspring. *Clinical and Experimental Allergy*.

[9] Lundholm C, Örtqvist AK, Lichtenstein P, Cnattingius S, Almqvist C (2010). Impaired fetal growth decreases the risk of childhood atopic eczema: a Swedish twin study. *Clinical and Experimental Allergy*.

[10] Linneberg A, Simonsen JB, Petersen J, Stensballe LG, Benn CS (2006). Differential effects of risk factors on infant wheeze and atopic dermatitis emphasize a different etiology. *Journal of Allergy and Clinical Immunology*.

[11] Purvis DJ, Thompson JMD, Clark PM (2005). Risk factors for atopic dermatitis in New Zealand children at 3.5 year of age. *British Journal of Dermatology*.

[12] Benn CS, Wohlfahrt J, Aaby P (2004). Breastfeeding and risk of atopic dermatitis, by parental history of allergy, during the first 18 months of life. *American Journal of Epidemiology*.

[13] Ludvigsson JF, Mostrom M, Ludvigsson J, Duchen K (2005). Exclusive breastfeeding and risk of atopic dermatitis in some 8300 infants. *Pediatric Allergy and Immunology*.

[14] Leadbitter P, Pearce N, Cheng S (1999). Relationship between fetal growth and the development of asthma and atopy in childhood. *Thorax*.

[15] Sevelsted A, Bisgaard H (2012). Neonatal size in term children is associated with asthma at age 7, but not with atopic dermatitis or allergic sensitization. *Allergy*.

[16] Moore MM, Rifas-Shiman SL, Rich-Edwards JW (2004). Perinatal predictors of atopic dermatitis occurring in the first six months of life. *Pediatrics*.

[17] Mallen CD, Mottram S, Wynne-Jones G, Thomas E (2008). Birth-related exposures and asthma and allergy in adulthood: a population-based cross-sectional study of young adults in North Staffordshire. *Journal of Asthma*.

[18] Christiansen L, Frederiksen H, Schousboe K (2003). Age- and sex-differences in the validity of questionnaire-based zygosity in twins. *Twin Research*.

[19] Duffy DL, Inspector TD, Snieder H, MacGregor AJ (2000). The co-twin control study. *Advances in Twin and Sib-Pair Analysis*.

[20] Xu B, Pekkanen J, Järvelin M-R (2000). Obstetric complications and asthma in childhood. *Journal of Asthma*.

[21] Metsälä J, Kilkkinen A, Kaila M (2008). Perinatal factors and the risk of asthma in childhood—a population-based register study in Finland. *American Journal of Epidemiology*.

[22] Räsänen M, Kaprio J, Laitinen T, Winter T, Koskenvuo M, Laitinen LA (2000). Perinatal risk factors for asthma in Finnish adolescent twins. *Thorax*.

[23] Räsänen M, Kaprio J, Laitinen T, Winter T, Koskenvuo M, Laitinen LA (2001). Perinatal risk factors for hay fever—a study among 2550 Finnish twin families. *Twin Research*.

[24] Källén B, Finnström O, Nygren KG, Otterblad Olausson P (2012). Asthma in Swedish children conceived by in vitro fertilisation. *Archives of Disease in Childhood*.

[25] Gilman SE, Gardener H, Buka SL (2008). Maternal smoking during pregnancy and children’s cognitive and physical development: a causal risk factor?. *American Journal of Epidemiology*.

[26] DaVeiga SP (2012). Epidemiology of atopic dermatitis: a review. *Allergy and Asthma Proceedings*.

[27] Schmitz R, Atzpodien K, Schlaud M (2012). Prevalence and risk factors of atopic diseases in German children and adolescents. *Pediatric Allergy and Immunology*.

[28] Silva LM, Jansen PW, Steegers EAP (2010). Mother’s educational level and fetal growth: the genesis of health inequalities. *International Journal of Epidemiology*.

[29] Hwang C-Y, Chen Y-J, Lin M-W (2010). Prevalence of atopic dermatitis, allergic rhinitis and asthma in Taiwan: a national study 2000 to 2007. *Acta Dermato-Venereologica*.

[30] Saeki H, Iizuka H, Mori Y (2005). Prevalence of atopic dermatitis in Japanese elementary schoolchildren. *British Journal of Dermatology*.

[31] Naldi L, Parazzini F, Gallus S (2009). Prevalence of atopic dermatitis in Italian schoolchildren: factors affecting its variation. *Acta Dermato-Venereologica*.

[32] Ballardini N, Kull I, Söderhäll C, Lilja G, Wickman M, Wahlgren CF (2013). Eczema severity in preadolescent children and its relation to sex, filaggrin mutations, asthma, rhinitis, aggravating factors and topical treatment: a report from the BAMSE birth cohort. *British Journal of Dermatology*.

[33] Friedman SA, Schiff E, Kao L (1997). Do twins mature earlier than singletons? Results from a matched cohort study. *American Journal of Obstetrics and Gynecology*.

